# ZIF-8/PI Nanofibrous Membranes With High-Temperature Resistance for Highly Efficient PM_0.3_ Air Filtration and Oil-Water Separation

**DOI:** 10.3389/fchem.2021.810861

**Published:** 2021-12-10

**Authors:** Yu Li, Dan Wang, Guanchen Xu, Li Qiao, Yong Li, Hongyu Gong, Lei Shi, Dongwei Li, Meng Gao, Guoran Liu, Jingjing Zhang, Wenhui Wei, Xingshuang Zhang, Xiu Liang

**Affiliations:** Shandong Provincial Key Laboratory of High Strength Lightweight Metallic Materials, Advanced Materials Institute, Qilu University of Technology (Shandong Academy of Sciences), Jinan, China

**Keywords:** polyimide, ZIF-8, nanofibers, high-temperature resistance, air filtration, oil-water separation

## Abstract

Air and water pollution poses a serious threat to public health and the ecological environment worldwide. Particulate matter (PM) is the major air pollutant, and its primary sources are processes that require high temperatures, such as fossil fuel combustion and vehicle exhaust. PM_0.3_ can penetrate and seriously harm the bronchi of the lungs, but it is difficult to remove PM_0.3_ due to its small size. Therefore, PM_0.3_ air filters that are highly efficient and resistant to high temperatures must be developed. Polyimide (PI) is an excellent polymer with a high temperature resistance and a good mechanical property. Air filters made from PI nanofibers have a high PM removal efficiency and a low air flow resistance. Herein, zeolitic imidazolate framework-8 (ZIF-8) was used to modify PI nanofibers to fabricate air filters with a high specific surface area and filtration efficiency. Compared with traditional PI membranes, the ZIF-8/PI multifunction nanofiber membranes achieved super-high filtration efficiency for ultrafine particles (PM_0.3_, 100%), and the pressure drop was only 63 Pa. The filtration mechanism of performance improvement caused by the introduction of ZIF-8/PI nanofiber membrane is explored. Moreover, the ZIF-8/PI nanofiber membranes exhibited excellent thermal stability (300 C) and efficient water–oil separation ability (99.85%).

## 1 Introduction

Air and water pollution due to rapid industrialization and urbanization poses a serious threat to the ecological environmental and human health (Wang B et al., 2015; Lv et al., 2019). Air pollution mainly refers to the presence of airborne particulate matter (PM), which mostly comes from combustion reactions, such as combustion of fossil fuel, biomass, and garbage (Liang et al., 2019). PM pollutants with an aerodynamic diameter of less than or equal to 2.5 μm (PM_2.5_) are the major hazardous substances because they can block the alveoli and damage the lungs (Li and Yin, 2019). In particular, PM_0.3_, owing to its smaller particle size than other PM, it can cause greater harm that may lead to cardiovascular diseases, respiratory disease, diabetes, and lung cancer (Mosanenzadeh et al., 2020; Wang Z et al., 2020; Zhang et al., 2020). PM has a complex composition and may carry as many as 791 bacterial genera, which can cause allergy and infection (Gao et al., 2019; Zhang et al., 2020). Aside from air pollution, the presence of oil in wastewater and the water pollution caused by spillage of oil–organic solvents also seriously threaten public health (Zhang W et al., 2013; Wang B et al., 2015; Li et al., 2019) Therefore, filter membrane materials that can used for PM air filtration and oil–water separation must be developed.

Nanofiber filter materials have a small fiber diameter, a high porosity, and a large specific surface area, which are characteristics that compensate for the shortcomings of conventional filter materials that cannot absorb PM_0.3_ with a small particle size (Li D et al., 2020; Zhang et al., 2020). Electrospinning technology is a simple method for obtaining continuous, uniform, and controllable nanofibers with various polymers (Doimoto and Greiner, 2018). Electrospinning, on one hand, is fast developing from a single-fluid process (Zhang et al., 2021) to coaxial (Ning et al., 2021), tri-axial (Wang et al., 2020a), side-by-side (Wang et al., 2020b), and other complicated processes (Aidana et al., 2021). On the other hand, more and more active components are encapsulated into electrospun nanofibers through chemical or physical methods to take advantages of the physical properties of electrospun nanofiber mats. As a high-performance engineering polymer, polyimide (PI) has been extensively explored (Liu et al., 2015). Owing to its excellent mechanical properties, thermal stability, low dielectric constants, and insulation, it can be used in various applications, such as gas separation, oil-water separation, and cell separation (Miao et al., 2013; Zhang R et al., 2016; Chen et al., 2017; Bai et al., 2019). Metal-organic frameworks (MOFs) are composed of metal ions or ion clusters connected by organic ligands (Ma et al., 2019), which are widely used in gas separation (Dai et al., 2018), filtration (Lai et al., 2016), catalysis (Wang et al., 2015a), and sensing (Wang et al., 2015b) because of their high specific surface area, high porosity, and adjustablility (Li T. T et al., 2020). Zeolitic imidazolate framework-8 (ZIF-8) is one of the most widely used MOFs. Owing to its special tetrahedral skeleton structure, ZIF-8 not only has a high specific surface area and porosity but also has excellent thermal stability and chemical stability (Dai et al., 2018). The application of ZIF-8 in powder form in filtration is limited. Therefore, PI nanofibers doped with ZIF-8 nanoparticles can harness this MOF’s special open metal position, functional group, and surface charge to achieve highly efficient air filtration (Hao et al., 2019). Moreover, the introduction of ZIF-8 can change its hydrophobicity for oil-water separation.

Three methods for loading ZIF-8 on nanofiber membranes are currently available, namely, *in-situ* growth method (Xu et al., 2018; Wang et al., 2019), impregnation method (Tao et al., 2013; Ren et al., 2019), and co-spinning method (Dai et al., 2016; Zheng et al., 2020). In *in-situ* growth method, the ZnO seed layer is deposited on the fiber surface, and then ZnO is converted into ZIF-8 by *in-situ* solvothermal growth. Li et al. (Xu et al., 2018) prepared a ZIF-8 seed on PVDF/ZnO membranes by activation step and then expanded it in the growth step to separate water from oil solution. However, this method involves numerous steps, which compromise the repeatability and stability of experiments (An et al., 2016). Impregnation method involves a simple preparation process, but controlling the size and distribution of ZIF-8 grains via this method is difficult (Bechelany et al., 2015). Co-spinning method has the unique ability to make ZIF-8 nanoparticles evenly disperse in nanofibers, and load contents are easy to control via this method. The most important advantage of co-spinning method is that one-step electrospinning is easy to operate, controllable, and repeatable (Ostermann et al., 2011). In addition, the most significant advantage is that ZIF-8 is directly doped in PAA precursor solution. The semi embedded structure makes ZIF-8 stably load the interior and surface of PI nanofiber membrane. Hao et al. (2019) prepared soluble PI/ZIF-8 nanofiber membranes for air filtration *via* electrospinning. By contrast, a previous work (Dai et al., 2016) prepared PI nanofiber membranes by using the electrospinning precursor polyamide acid (PAA) solution on the gate receiver. This innovation has lowered the cost of PI synthesis, broaden its application range, and increased its filtration efficiency (the maximum filtration efficiency of PM_0.3_ was 100%).

Here, we developed a low-cost and a highly efficient material for filtering PM, which can be applied to high-temperature industrial waste filtration without affecting the structure and morphology of filtration materials. The unit price of polyimide powder required for the preparation of PI nanofibers by one-step method is as high as 17.2 yuan/g, while the average unit price of PMDA and ODA used in this study is only 1.08 yuan/g, which shows that the material cost developed in this study is low. The nanofiber diameter, porosity, filtration, and separation performance of the PI nanofibers membrane can be controlled by the loading contents of ZIF-8 nanoparticles (Lin et al., 2016; Ji et al., 2019; Dou et al., 2020; Wu et al., 2021). To address the attendant problems in air filtration and oil–water separation, we prepared herein highly efficient ZIF-8/PI nanofiber membranes via a simple co-spinning method. Moreover, the PI nanofiber membranes could be modified into superhydrophobic and superlipophilic membranes, a property that is not only conducive to the filtration of PM particles but can also be applied to oil-water separation. The filter membranes can be used for oil-water separation because the positive charge carried by ZIF-8 can interact with the negative charge carried by the oil drop to enhance its lipophilicity, and its superhigh specific surface area leads to hydrophobic interactions, thereby improving filtration efficiency.

## 2 Experimental Section

### 2.1 Materials

Pyromellitic dianhydride (PMDA), 4,4-oxidianiline (ODA), N,N-dimethylacetamide (DMAc), petroleum ether (C_5_H_12_), n-hexane (C_6_H_14_), oil red O (C_26_H_24_N_4_O), methylene blue (MB, C_16_H_18_C_l_N_3_S), and sodium chloride (NaCl) were purchased from Shanghai Macklin Biochemical Co., Ltd., Zinc nitrate hexahydrate [Zn(CH_3_COO)_2_] and 2-methylimidazole were bought from Aladdin (Shanghai, China). Dichloroethane (C_2_H_4_Cl_2_) and toluene (C_6_H_5_CH_3_) were procured from Sinopharm Chemical Reagent (Shanghai, China). All of these chemicals were used as received. All solutions were prepared with ultrapure water supplied by a water-purifying device in our laboratory.

### 2.2 Synthesis of ZIF-8

Zn(CH_3_COO)_2_ (175 mg) and 2-methylimidazole (263 mg) were dissolved in methanol (40 ml) by stirring at room temperature for 5 min and standing for 24 h. The white solid powder was centrifuged three times with methanol and dried at 120°C. The Raman spectra of the prepared powder samples (Supplementary Figure S1) were the same as those of known structural data, indicating that the powder obtained was ZIF-8. As shown in Supplementary Figure S2A, the synthesized ZIF-8 particles had a uniform dodecahedral structure. The particle size of the ZIF-8 precursor was approximately 280 nm (Supplementary Figure S2B).

### 2.3 Preparation of PI Nanofiber Membranes

PI nanofibers were synthesized *via* a two-step method (Supplementary Figure S3). ODA and PMDA were weighed in equal molar ratio and dissolved in DMAC to form 20wt% PAA precursor solution. When preparing the solution, ODA was first dissolved in DMAC, and then PMDA was added into the flask 8–10 times after complete dissolution. At the end of feeding, the solution was continuously and quickly stirred for 1 h to ensure that the monomer definitively indicated the formation of PAA. At the end of the reaction, the solution was allowed to stand for 12 h.

The prepared solution was electrospun on a metal mesh fixed on the surface of the drum shaped receiver. The voltage was set at 20 kV, the rotation speed was 200 r/min, and the steady flow rate was 0.95 ml/h. The material was extruded from an 18 G needle tip. After electrospinning, PAA was imidized into PI nanofibers by annealing at 350°C in an air muffle furnace (Supplementary Figure S4, FT-IR spectroscopy showed imidization).

### 2.4 Preparation of ZIF-8/PI Nanofiber Membranes

The precursor solution of ZIF-8/PAA was prepared via electrospinning. First, ZIF-8 was dissolved in DMAC, and the same experimental steps outlined in the previous section were followed. By controlling the mass ratio of ZIF-8 to PMDA, ODA, which was the precursor solution with doping amounts of 0, 1, 2, and 3%, was prepared. The same parameters of electrospinning and annealing mentioned in the preceding section were used.

### 2.5 Air Filtration Performance

The filtration efficiency and pressure drop of the nanofiber membranes were measured using a comprehensive performance test instrument for filter materials (Supplementary Figure S5). This instrument was connected to the PM particle emission source, and it regulated the air flow velocity through an air pump. PM particles with diameters ranging from 300 nm to 10 μm were supplied by neutral monodispersed solid sodium chloride aerosol, and filtration efficiency was calculated by a particle counter connected at both sides before and after filtration. The test was conducted at a steady flow rate of 4–14 L/min, and the effective area of the air inlet of the filter was 200.9 cm^2^.

### 2.6 Oil-Water Separation Tests

The oil–water separation performance of the membranes was tested using a simple oil–water separator. The prepared membranes were fixed between two sealable and linkable glass devices, and a conical bottle was placed at the bottom as a receiving container. When the mixture of water and oil (1/1 volume ratio) was directly poured into the upper glass container, the oil passed through the membrane and the water remained.

### 2.7 Characterizations

The morphology of the nanofiber membranes was examined via SEM. The structure of PI and the imidization process of PAA were analyzed via Fourier Transform Infrared (FTIR) spectroscopy. The composition of the ZIF-8/PI nanofiber membranes was analyzed *via* X-ray Photoelectron Spectroscopy (XPS) and Raman spectroscopy. The thermal stability of the membranes was tested via Thermogravimetric Analysis (TGA) at the heating rate of 5°C/min. The mechanical properties of the membranes were tested using a tensile tester. The porosity and specific surface area of the membranes were measured *via* Brunauer-Emmett-Teller (BET) analysis. The filtration efficiency and pressure drop of the membranes were tested by a mask tester (G506). The device used for the oil-water separation experiment was self-assembled.

## 3 Results and Discussion

### 3.1 Characterization of the ZIF-8/PI Nanofiber Membranes

**FIGURE 1:**
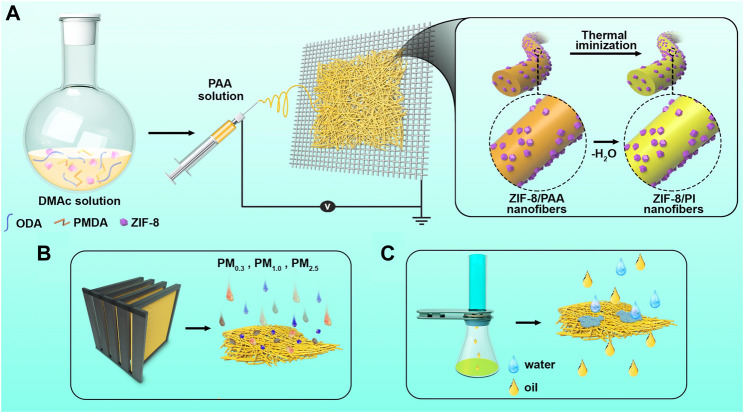
Schematic of the procedure for preparing ZIF-8/PI electrospun nanofiber membranes and their applications. **(A)** Schematic of the membranes prepared by the two-step electrospinning method. **(B)** Air filtration of the membranes. **(C)** Water–oil separation.

**FIGURE 2:**
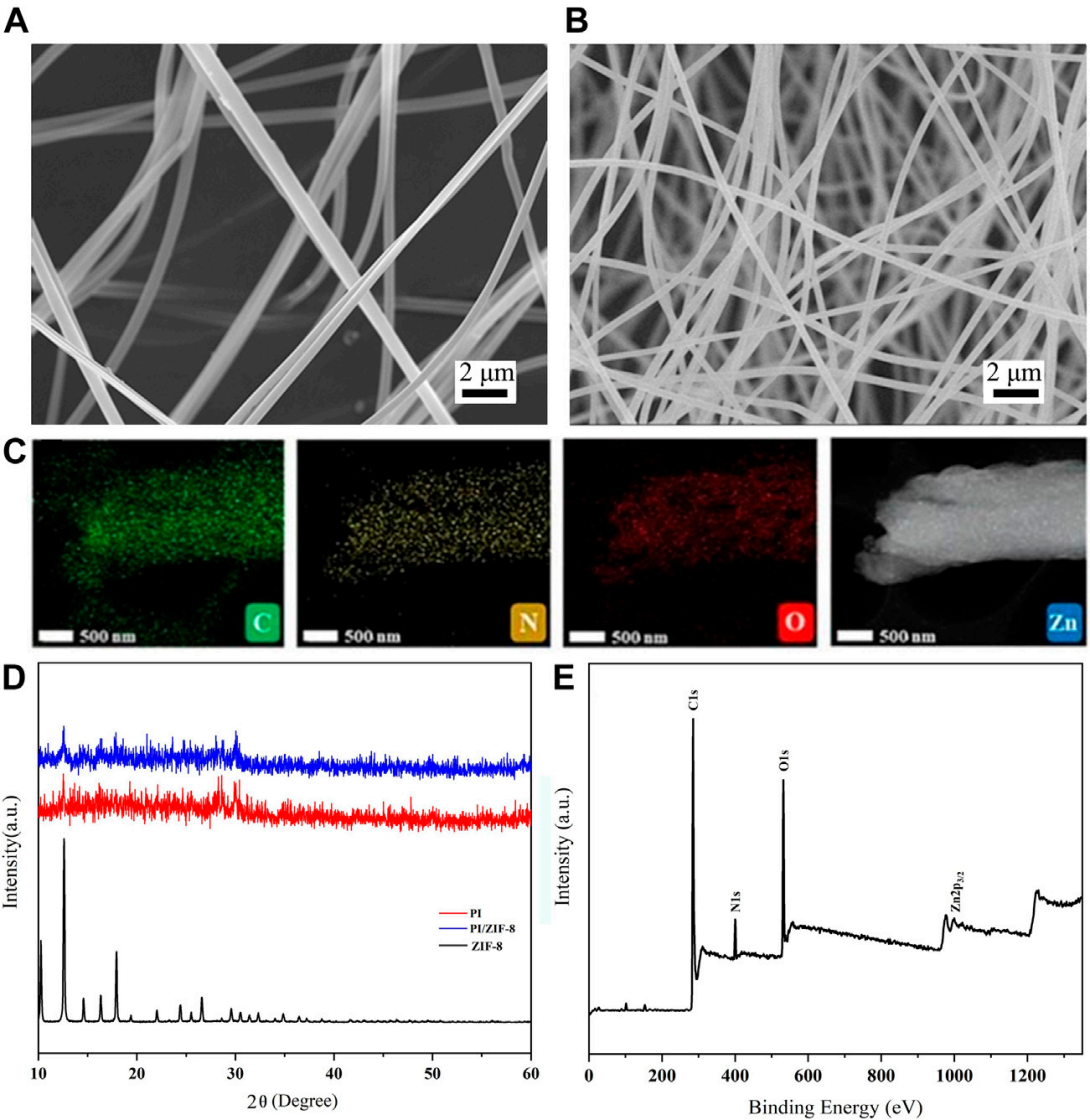
Scanning electron micrographs of the PI-ZIF nanofibers **(A)** PI and **(B)** ZIF-8-3/PI. **(C)** C, N, O, and Zn element distribution of ZIF-8/PI nanofibers. **(D)** X-ray diffraction patterns of the ZIF-8, PI, and ZIF-8-3/PI nanofiber membranes. **(E)** X-ray photoelectron spectroscopy images of the ZIF-8-3/PI nanofiber membranes.

Figure 1A shows the method for preparing the ZIF-8/PI nanofiber membranes and their application. The ZIF-8/PI nanofiber membranes were obtained by electrospinning the ZIF-8/PAA nanofiber membranes and via thermal imidization (Figure 1A). Owing to the doping of ZIF-8 nanoparticles, the specific surface area and porosity of ZIF-8/PI nanofiber membranes increased, improving the air filtration and oil-water separation properties (Figures 1B,C). ZIF-8-1/PI, ZIF-8-2/PI, and ZIF-8-3/PI were used to represent the loading content of 1, 2, and 3%, respectively, to distinguish the samples with different loading contents of ZIF-8. As shown in Figure 2A, the diameter of the as-prepared PI nanofibers was approximately 340 nm and their nanogrid pores were large. The surface morphology of ZIF-8 nanoparticles only marginally changed and was slightly rough. As shown in Figure 2B, the ZIF-8/PI nanofiber diameter substantially decreased to about 300 nm, and the nanogrid structure was more compact. The X-Ray Diffraction (XRD) patterns of the ZIF-8-3/PI nanofiber membranes were similar to those of pure PI nanofiber membranes, but the peaks of the ZIF-8/PI nanofiber membranes at 12.75° and 19.53° were enhanced, which corresponded to the position of the characteristic peak of ZIF-8 (Wang et al., 2015a). Furthermore, the element distribution of the full elements of C, N, O, and Zn of the ZIF-8/PI nanofibers (Figure 2C) indicated that ZIF-8 nanoparticles were evenly distributed in the PI nanofibers. The XRD patterns of the ZIF-8-3/PI nanofiber membranes contained the characteristics of both ZIF-8 and pure PI, indicating that ZIF-8 nanoparticles were successfully embedded into the PI nanofiber membranes (Figure 2D). The XPS spectra (Figure 2E; Supplementary Figure S6) indicated that ZIF-8 nanoparticles were introduced in the PI nanofibers successfully.

### 3.2 PM_0.3_ Filtration Performance of the PI Nanofiber Membranes

**FIGURE 3:**
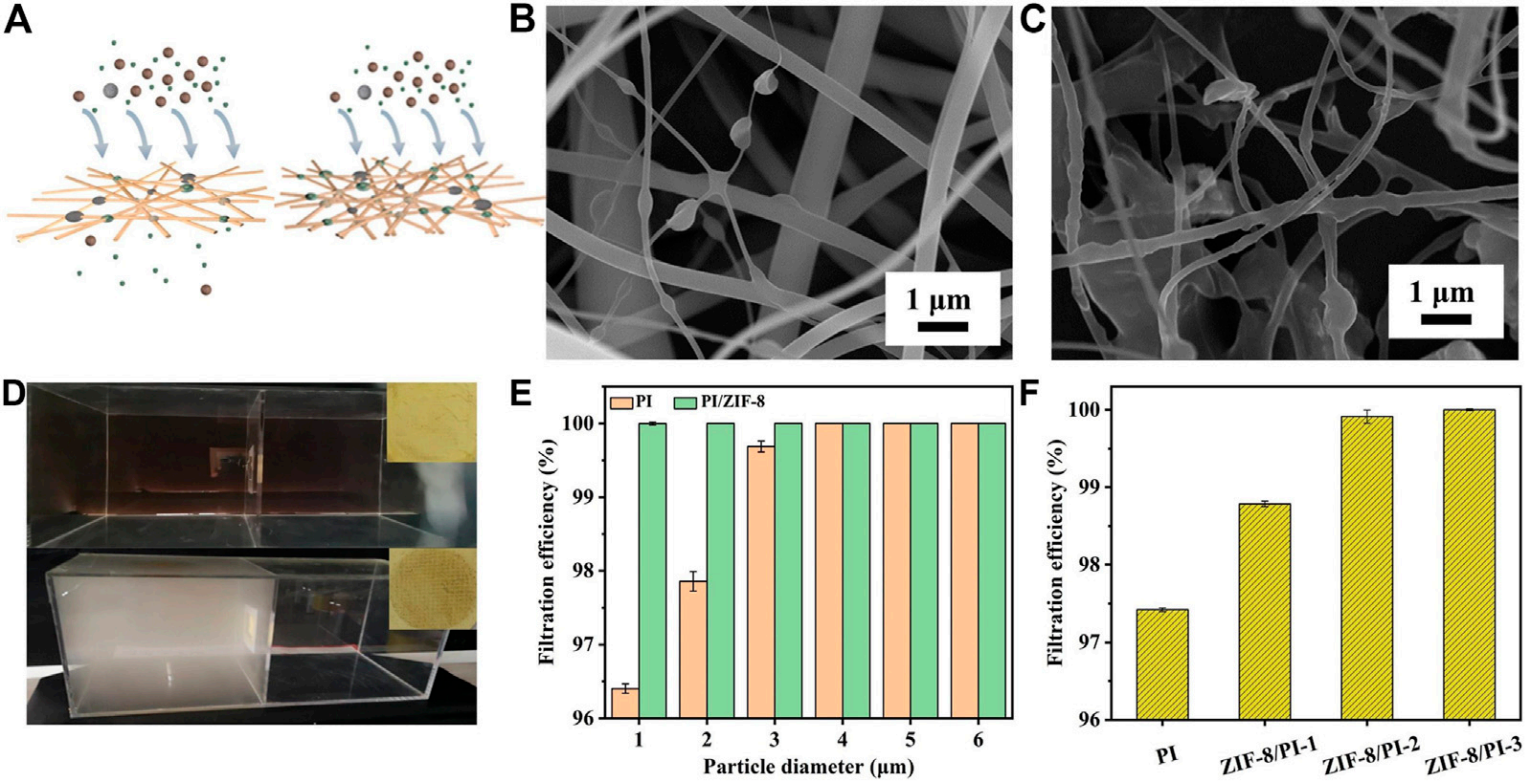
**(A)** Diagram of different fiber membranes before and after filtration. **(B)** Scanning electron micrograph (SEM) of the PI fiber membranes after filtration. **(C)** SEM of the ZIF-8-3/PI fiber membranes after filtration. **(D)** Digital image of the nanofiber filter membrane before and after filtering in the filtering device. **(E)** Comparison of the filtration efficiency of the PI and ZIF-8/PI-3 fiber membranes for PM_0.3_ filtration at the air flow rate of 6 L/min). **(F)** Effects of the PI nanofiber membranes with different ZIF-8 loadings on PM_0.3_ filtration efficiency.

Figure 3A shows the process of membrane filtration of PM particles. To obtain a large amount of filtered PM membranes, the device shown in Figure 3D was used for continuous long-term filtration, and incense was ignited in the left acrylic box to provide PM source. After adsorbing PM particles, the PI nanofiber membranes changed from light yellow to brown (Figure 3D, inset). Within the same filtration time, the filtration capacity of PM particles on PI and the ZIF-8-3/PI nanofiber membranes was obviously different. The dense grid structure of the ZIF-8-3/PI nanofibers was conducive to the filtration of PM particles. As shown in Figures 3B,C, the major PM particles were adsorbed on the fiber nodes, while the smaller porosity (Supplementary Figure S7), the more nodes (Zhang et al., 2019). Moreover, the introduction of ZIF-8 increased the specific surface area of the membranes, thus providing more filtration points for PM filtration (Hao et al., 2019). Figure 3E also directly shows that the introduction of ZIF-8 substantially improved the filtration efficiency of the membranes. The filtration efficiency of the PI nanofiber membranes for PM_0.3_ was only 96.38%, whereas that of the doped ZIF-8/PI nanofiber membranes could reach 100% (6 L/min). Furthermore, the smaller the diameter of PM particles was, the higher the filtration efficiency was. According to the comparative report, compared with the non-woven PI membrane in the previous research (Li et al., 2017), the filtration efficiency of ZIF-8/PI nanofiber membrane PI nanofiber membrane prepared in this study is considerably higher than that formed by multi-layer fiber composite, and it also has significant advantages in pressure drop (Wang et al., 2016). A comparison of the filtration efficiency of the different loading contents of ZIF-8 in the PI nanofiber membranes (Figure 3F), it can be seen that when the loading amount of ZIF-8 was lower than 3 wt%, the filtration efficiency of the PI nanofiber membranes gradually increased with the increase in ZIF-8 doping amount. However, excessive doping of ZIF-8 affected the conductivity of the precursor solution, which was not conducive to electrospinning. Therefore, the optimal doping ratio was 3 wt%.

**FIGURE 4:**
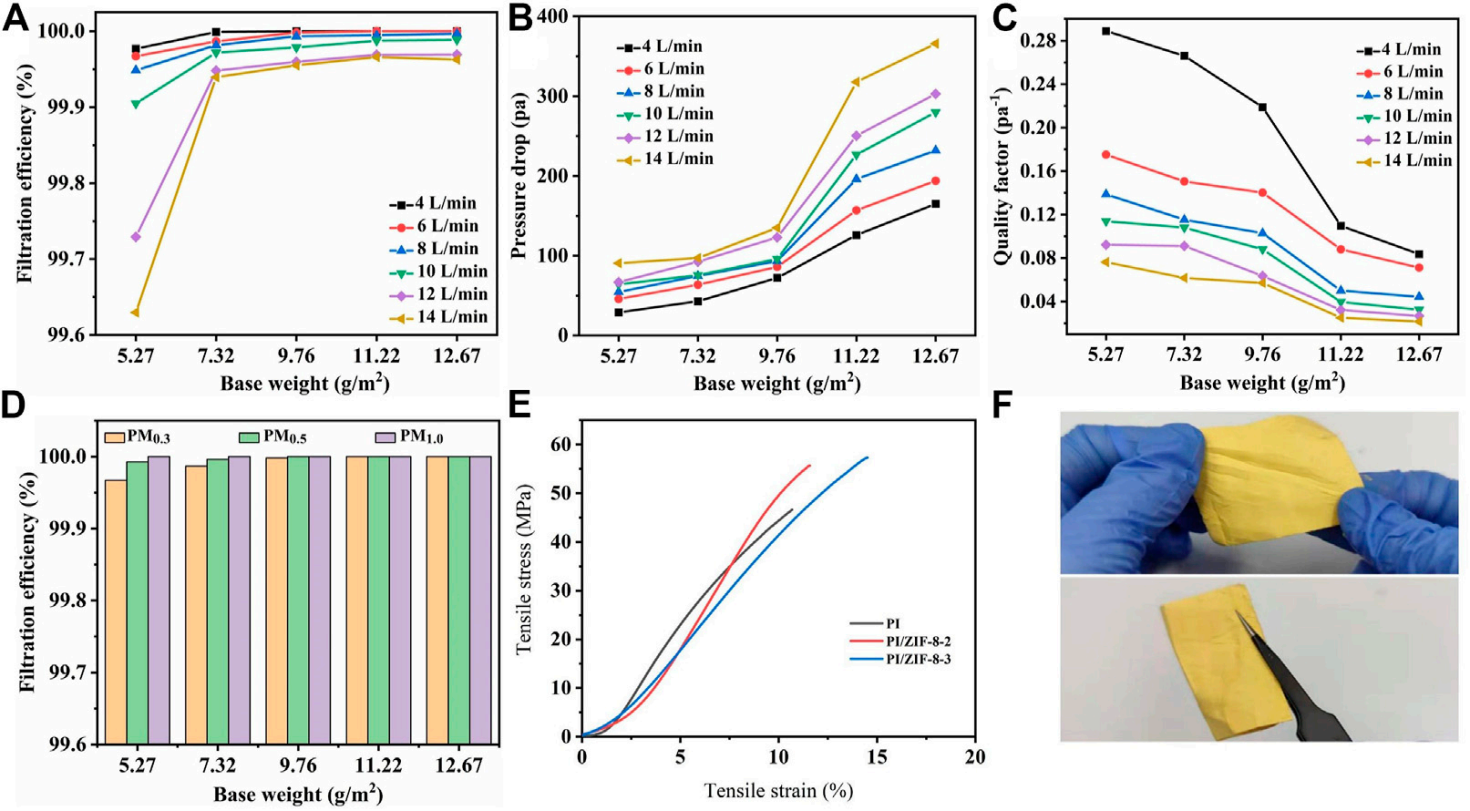
The filtration efficiency **(A)**, pressure drop **(B)**, and quality factor **(C)** of the ZIF-8-3/PI membranes varied with base weight and air flow rate. **(D)** Effects of different base weights of the ZIF-8/PI nanofiber membranes on the filtration efficiency of PM_0.3_, PM_0.5_, and PM_1.0_. **(E)** Stress–strain curves. **(F)** Rigidity and flexibility of the ZIF-8/PI nanofiber membranes.

As the base weight of the ZIF-8-3/PI nanofiber membranes increased, the filtration efficiency of PM_0.3_ considerably improved (Figure 4A) because the increase in base weight increased the thickness of the nanofiber membranes, thereby prolonging the contact time between PM and the fibers and enhancing the filtration efficiency. By contrast, the increase in air velocity reduced the contact time, which was not conducive to PM filtration. However, the increase in both base weight and air velocity also increased the pressure drop, which was not conducive to the service life of the membranes. The best base weight of the membranes was determined to be 7.32 g/m^2^. Moreover, the pressure drop was only 63 Pa at 14 L/min, which was considerably lower than that of traditional commercial filters (data). Furthermore, the filtration efficiency was as high as 99.9375%. When air velocity was reduced to 6 L/min at this weight, the filtration efficiency of PM_0.3_ could reach 100% (Figure 4B). Quality factor (*Q*_*F*_) was used to evaluate the comprehensive performance of the filter materials, which is defined as follows (1):

$$Q_{F}=\frac{-\ln\left(1-\eta \right)}{\Delta P}\text{,}$$ (1)

where *η* is filtration efficiency, and Δ*P* is pressure drop. According to Eq. 1, pressure drop is an important factor affecting *Q*_*F*_. The lowest pressure drop was at 4 L/min. When the base weight was 5.27, 7.32, 9.76, 11.22, and 12.67 g/m^2^, the values of *Q*_*F*_ were 0.2888, 0.2661, 0.2189, 0.1096, and 0.0837, respectively (Figure 4C). A high *Q*_*F*_ value also indicated a high PM interception performance. The ZIF-8/PI nanofiber membranes had a very high filtration efficiency (>99.9%) for all PM particles (PM_0.3–10_), even when the base weight was only 5.27 g/m^2^ (Figure 4D). With the introduction of ZIF-8, the tensile strength and elongation of the membrane increased slightly. With the increase of ZIF-8 content, it is directly proportional to the improvement of mechanical properties (Figure 4E). Aside from their high tensile strength, the ZIF-8-3/PI nanofiber membranes also had a good flexibility and could be bent at will (Figure 4F).

Apart from physical filtration, the air filtration mechanism of ZIF-8/PI nanofiber membrane include: 1) The positive charge on the surface of ZIF-8 nanoparticles can polarize the surface of PM particles, making it easier to interact with nanofibers, thus improving the ability of the membrane to capture PM particles (Ma et al., 2019; Dou et al., 2020; Wu et al., 2021). 2) The high specific surface area of ZIF-8 nanoparticles will improve the specific surface area of the PI nanofiber membrane, which is helpful to improve the surface energy of the membrane and increase the adsorption efficiency (Lin et al., 2016). 3) The essence of adhesion is that when air flows around nanofibers, a certain force should be responsible for fixing the tiny particles in the air (Gong et al., 2015). The results show that although the adhesion effect is widespread in nanofibers, the fiber diameter has a significant effect on the adsorbability (Zhang Y et al., 2016). After the introduction of ZIF-8 nanoparticles, the diameter of PI nanofibers reduced, and the slightly rough surface morphology provides more adsorption sites for PM particles, resulting in a higher adhesion to PM particles (Miao et al., 2013; Liu et al., 2015; Ji et al., 2019).

### 3.3 Thermal Stability of the ZIF-8/PI Nanofiber Membranes

**FIGURE 5:**
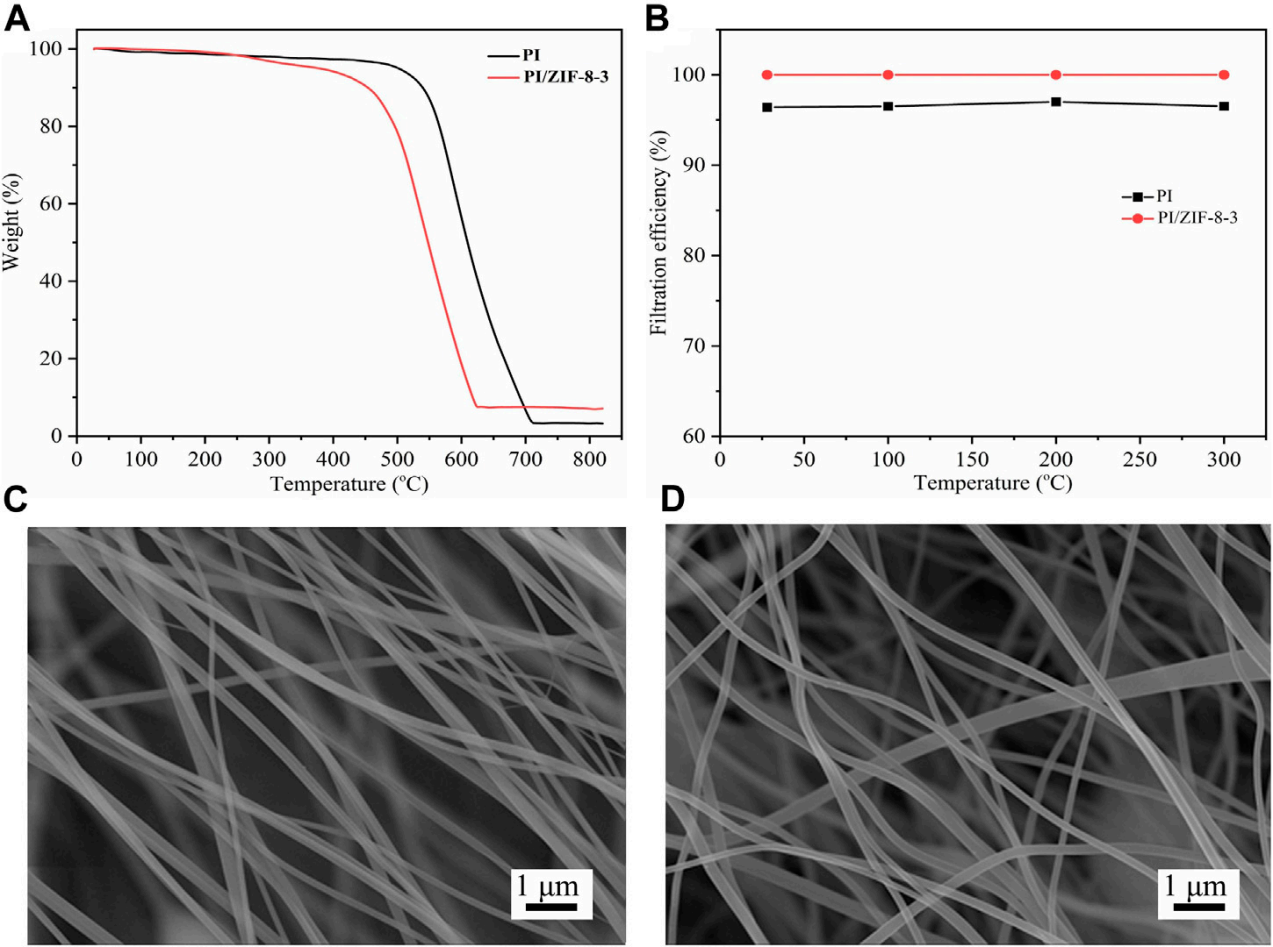
**(A)** Thermogravimetric curves of PI and the ZIF-8-3/PI membranes. **(B)** Filtration efficiency of the PI nanofiber membranes and the ZIF-8-3/PI nanofiber membrane to PM_0.3_ after being treated at different temperatures for 2 h. Scanning electron micrographs of the ZIF-8/PI nanofiber membranes before **(C)** and after **(D)** heat treatment (300°C).

Among the advantages of the PI nanofiber membranes was the fact that they could be used in high-temperature gas filtration. The resistance of the ZIF-8-3/PI nanofiber membranes to high temperatures was tested *via* TGA. As shown in Figure 5A, the change trend of the two membranes was roughly the same from room temperature up to 800°C. The pyrolysis of the ZIF-8/PI nanofiber membranes begun at 450°C because the pyrolysis temperature of ZIF-8 was near that temperature. By comparison, the pyrolysis of the PI nanofiber membranes begun at 500°C (Yang et al., 2020). Although the introduction of ZIF-8 reduced the initial pyrolysis temperature of the membranes, they remained suitable for high-temperature gas filtration (300°C). In practical filtration applications, the membranes were heat-treated at 100°C, 200°C, and 300°C for 2 h, and then their filtration efficiency was tested. Results showed that the filtration efficiency of both the PI nanofiber membranes and the ZIF-8/PI nanofiber membranes for PM_0.3_ did not change after heat treatment (Figure 5B). The morphology of the ZIF-8/PI nanofiber membranes did not change after heat treatment at 300°C (Figures 5C,D), also proving that the ZIF-8/PI nanofiber membranes were the thermally stable.

### 3.4 Oil–Water Separation Performance of the PI/ZIF-8 Nanofiber Membranes

**FIGURE 6:**
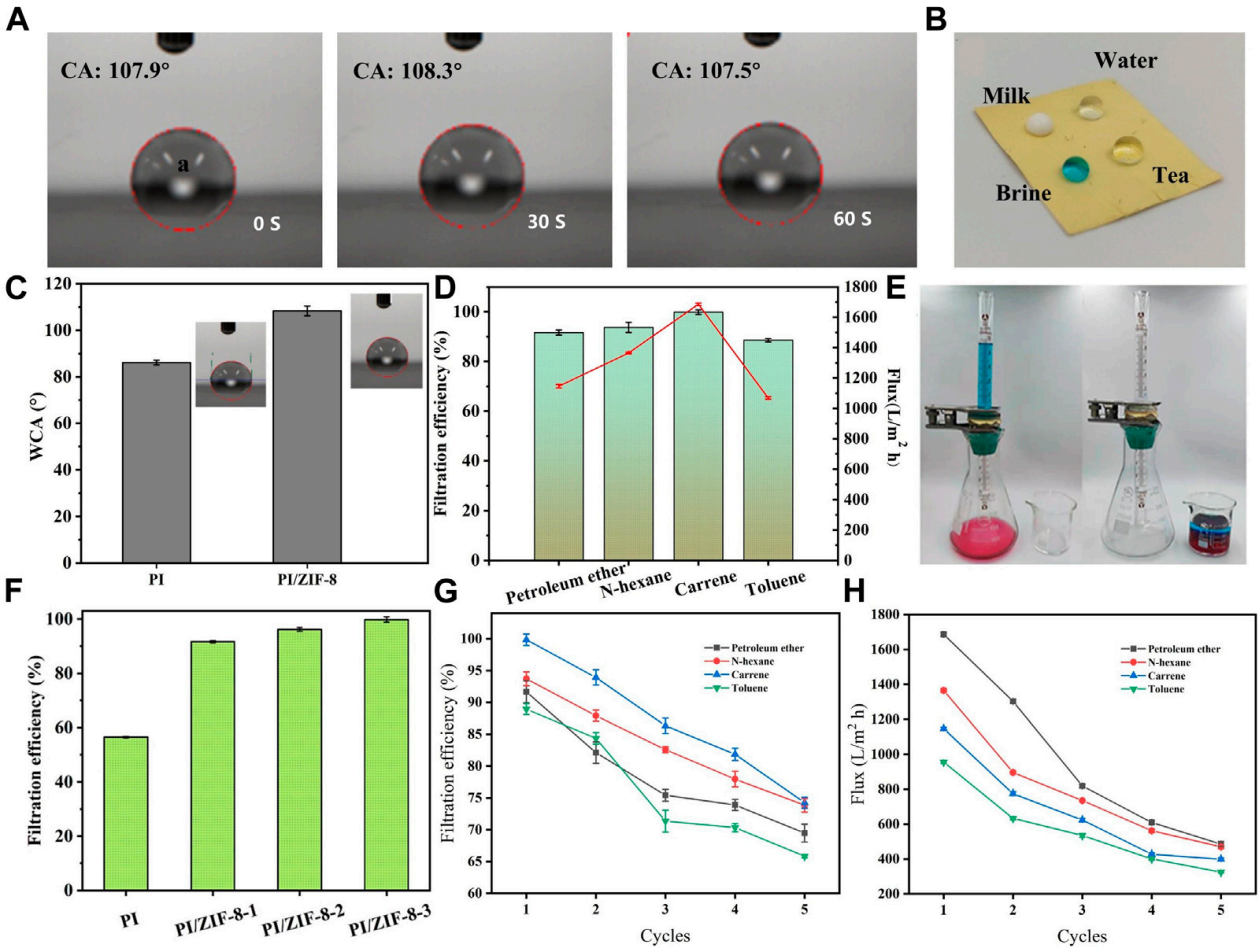
**(A)** Contact angles of hydrophobic ZIF-8/PI nanofiber membranes. **(B)** Droplets of different pollution levels can be kept spherical. **(C)** The water-contact angles of both the PI nanofiber membranes and the PI/ZIF-8 nanofiber membranes were measured. **(D)** Water–oil separation efficiency. **(E)** Filter unit diagram. **(F)** The filtration efficiency of dichloromethane by PI membrane with different doping amount of ZIF-8. **(G)** Circulating filtration efficiency. **(H)** Circulating filtered oil flux.

The wettability of membranes to water and oil is an important prerequisite for their application to oil–water separation. It can be seen from capillary utility that the lipophilic membrane will become more lipophilic with the increase of surface roughness (Cai et al., 2017). At the same time, the introduction of ZIF-8 will significantly reduce the pore size of the membrane (Supplementary Figure S7), and the smaller pore size is also conducive to improve the oil-water separation efficiency (Xu et al., 2018; Wu et al., 2021). The PI/ZIF-8 nanofiber membranes had a high surface roughness because of the introduction of ZIF-8, which changed the hydrophobicity of the PI nanofiber membranes (Zhang F et al., 2013). The original PI membrane was transformed into a hydrophobic PI membrane, and the contact angle was changed from 87° to 107° (Figure 6C). This is also due to the hydrophobicity of ZIF-8. The water droplets on the surface of the PI/ZIF-8 nanofiber membranes were spherical and could last for 60 s (Figure 6A). The PI/ZIF-8 nanofiber membranes had good self-cleaning performance because they had a great contact angle to all kinds of common liquids in daily life, such as milk, water, brine, and tea (Figure 6B).

An experimental separation device (Figure 6E) was designed to evaluate the oil–water separation performance of the membranes. Separation performance was calculated by Eq. 2 as follows:

$$\eta =\frac{M_{1}}{M_{0}}\times 100\%\text{,}$$ (2)

where *M*_*1*_ and *M*_*o*_ represent the quality of oil before and after separation, respectively. Owing to the superlipophilic property of the ZIF-8/PI nanofiber membranes, they could achieve highly efficient separation for various oily reagents. Separate 10 ml water oil mixed solution of oily reagents Petroleum ether, N-hexane, petroleum ether carrene and toluene respectively. According to Figure 6D, the filtration efficiency of dichloromethane can be as high as 99.85%, while that of other oily reagents can also be as high as 91.66, 93.71, 88.56%. As shown in Figure 6F, taking carrene as the filtration object, with the increase of ZIF-8 doping, the filtration efficiency is significantly improved, from 56.54% of pure PI to 99.85% of ZIF-8/PI. The different filtration efficiency may be due to the different viscosity of different oily reagents and the different blockage degree of fiber pores. Reflected in the oil flux is that the oil flux is positively correlated with the filtration efficiency. In order to study the fouling of oil on the membrane, the circulating performance of ZIF-8/PI membrane was tested, and it was found that its circulating filtration performance needs to be improved. Compared with the oil flux data in Figure 6H, with the increase of test times, the oil flux decreases, which also directly reflects the reduction of filtration efficiency, as shown in Figure 6G. Therefore, it is necessary to further improve the recoverability and circulating filtration performance of the membrane in the follow-up research. Therefore, the ZIF-8/PI nanofiber membranes can be widely used in the separation of light oil–water mixture (ρ_oil_ < ρ_water_).

## 4 Conclusion

PI/ZIF-8 nanofiber membranes for air filtration and oil–water separation were developed in this work, and its filtration mechanism was studied. The ZIF-8/PI nanofiber membranes obtained *via* doping and the co-spinning method exhibited excellent synergistic properties. Owing to the versatility of ZIF-8, the membranes could achieve super highly efficient filtration (PM_0.3_, 100%; 6 L/min) and highly efficient oil-water separation (99.85%). In addition, it’s a new developing idea to electrospin the ZIF-8 precursor on the polymer nanofibers using a coaxial process to surficializate the functional particles. The excellent comprehensive properties of the ZIF-8/PI nanofiber membranes, including their super highly efficient low-pressure air filtration, oil-water separation, and thermodynamic stability, indicate that they have broad industrial application prospects.

## Data Availability

The original contributions presented in the study are included in the article/Supplementary Material, further inquiries can be directed to the corresponding authors.
