# Supplementary material for: ZIF-8/PI Nanofibrous Membranes With High-Temperature Resistance for Highly Efficient PM0.3 Air Filtration and Oil-Water Separation
Source: Front Chem. 2021 Dec 10;9:810861. doi: 10.3389/fchem.2021.810861 (PMC8702621; doi:10.3389/fchem.2021.810861)
Supplement: Supplementary file 1 [file DataSheet1.docx]

**Supporting Information**

**ZIF-8/PI Nanofibrous Membranes with High-Temperature Resistance for Highly Efficient PM_0.3_ Air Filtration and Oil-Water Separation**

Yu Li, Dan Wang, Guanchen Xu, Li Qiao, Yong Li, Hongyu Gong, Lei Shi, Dongwei Li, Meng Gao, Guoran Liu, Jingjing Zhang, Wenhui Wei, Xingshuang Zhang^*^ and Xiu Liang^*^

*Shandong Provincial Key Laboratory of High Strength Lightweight Metallic Materials, Advanced Materials Institute, Qilu University of Technology (Shandong Academy of Sciences), Jinan 250014, P. R. China.*

^*^Corresponding authors: Xingshuang Zhang, Xiu Liang

Email addresses: [xszhang@qlu.edu.cn](mailto:xszhang@qlu.edu.cn)

Email addresses: [xliang@sdas.org](mailto:xliang@sdas.org)


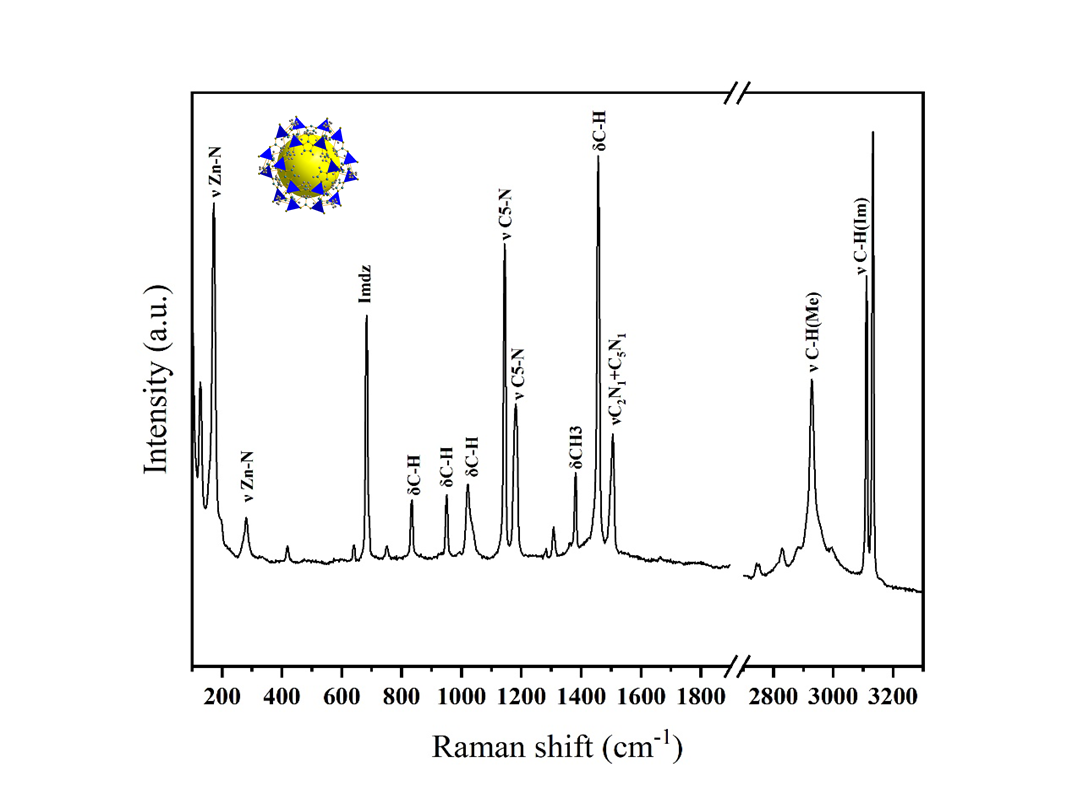


Figure S1. Raman spectra of as-prepared ZIF-8 nanoparticles.


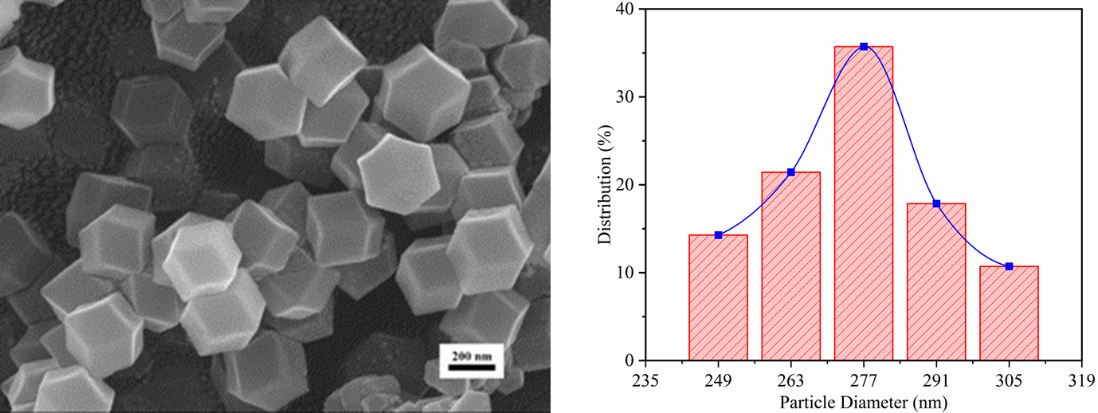


Figure S2. SEM image（a）and particle size distribution （b） of ZIF-8 nanoparticles.


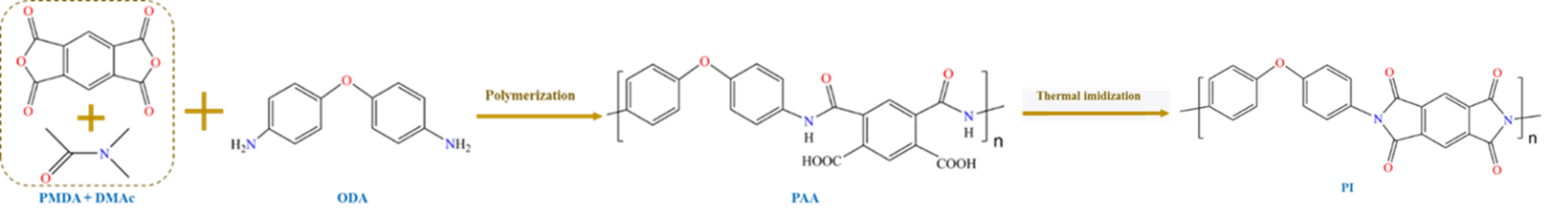


Figure S3. Two step preparation of Polyimide (PI).


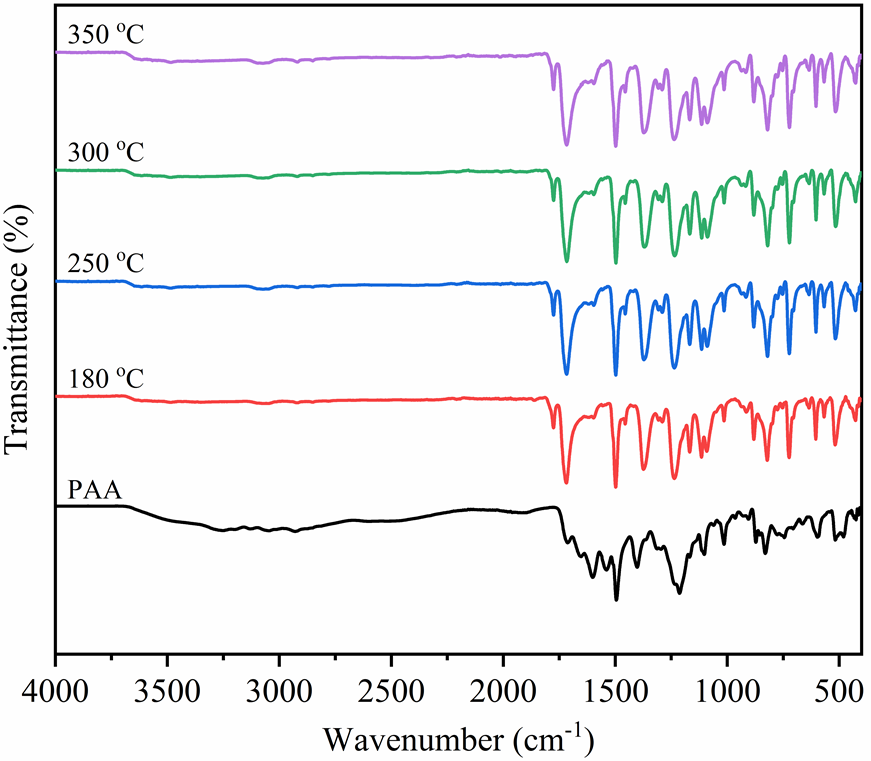


Figure S4. FT-IR spectra of PAA nanofibers obtained by thermal imidization at different temperatures.


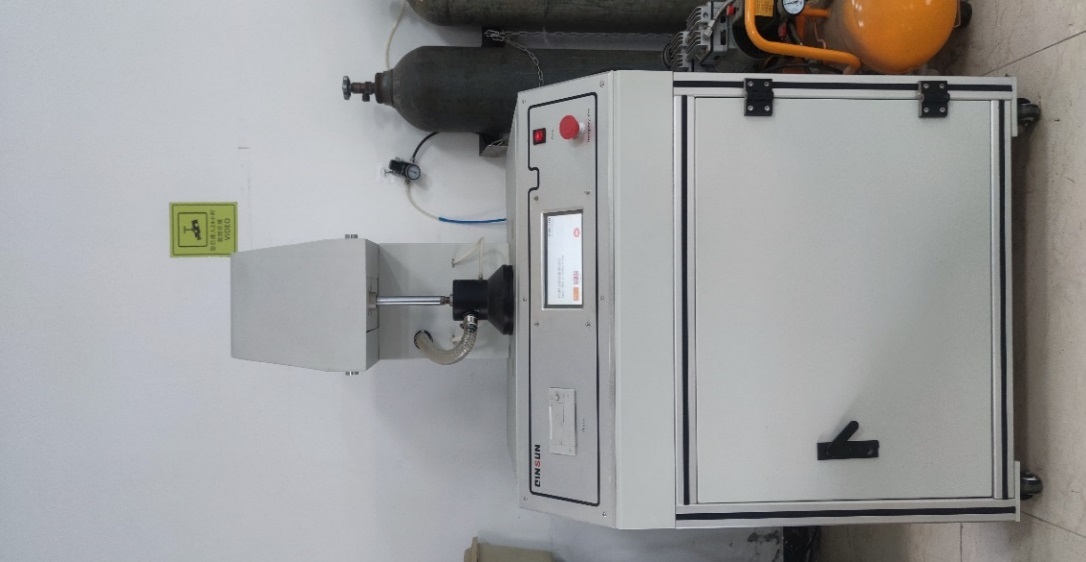


Figure S5. Comprehensive performance test instrument of filter materials.


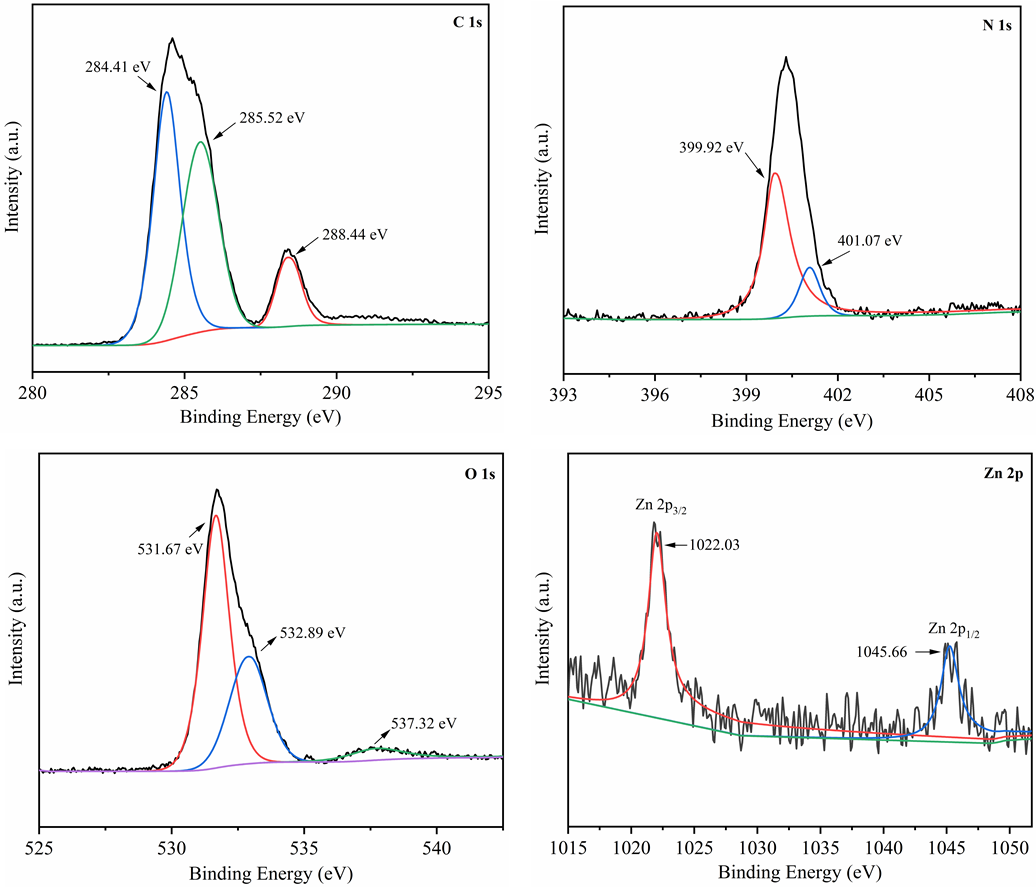


Figure S6. XPS spectra of C1s, N1s, O1s and Zn2p of as-prepared ZIF-8/PI nanofiber membrane.


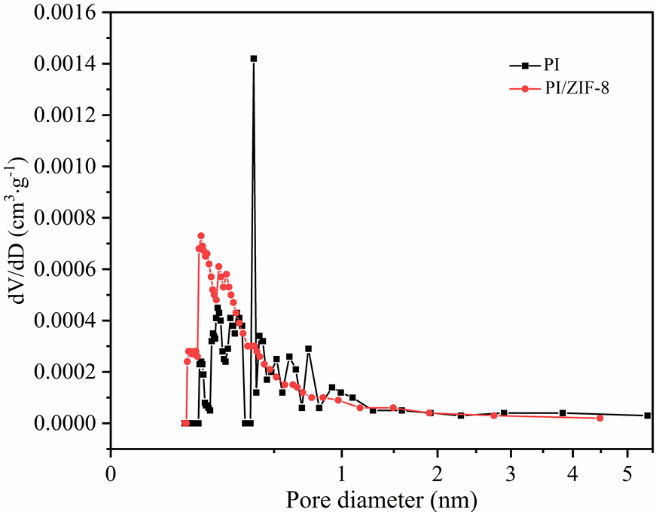


Figure S7. Pore volume distribution of PI nanofiber membrane and ZIF-8/PI nanofiber membrane.
